# Quality assurance for point-of-care testing: Ethiopia’s experience

**DOI:** 10.4102/ajlm.v5i2.452

**Published:** 2016-10-17

**Authors:** Adisu Kebede, Yenew Kebede, Adino Desale, Achamyeleh Mulugeta, Zelalem Yaregal, Atsbeha Gebreegziabxier, Yared Tedla, Clement Zeh, Gonfa Ayana

**Affiliations:** 1 Ethiopian Public Health Institute, Ethiopia; 2 Centers for Disease Control and Prevention, Ethiopia Laboratory Branch, Ethiopia

## HIV epidemiology in Ethiopia

In 2015, there were an estimated 729 517 people living with HIV in Ethiopia, including 95 094 children (aged 0–14 years), according to the 2015 Estimation and Projection Package/Spectrum modelling. HIV prevalence at the national level is 4.2% among urban populations and 0.6% rural populations; by gender, HIV prevalence is 1.52% among women and 1.0% among men.^1^ The HIV epidemic in Ethiopia is primarily associated with proximity to major transport corridors and concentrated in urban areas, where prevalence is 5.2% in cities with a population above 50 000 people compared with 2.8% in smaller cities and 0.6% in rural areas.^2^

Considerable progress has been made over the last decade in scaling up access to testing worldwide. The Federal Ministry of Health of Ethiopia adapted and started implementation of programmes to achieve the ambitious UNAIDS 90-90-90 goal, whereby 90% of HIV-positive people are diagnosed by 2020, 90% of HIV-positive patients are on antiretroviral therapy, and 90% of HIV-positive patients achieve viral suppression.^3,4^ However, the biggest challenges to achieving the 90-90-90 goals in Ethiopia are the first two goals. Currently, 40% of infected people are unaware of their HIV status, with 12.1% of those identified as HIV-positive being neither in care nor on antiretroviral therapy. There is also very limited information available on viral suppression. Therefore, closing these gaps would require some innovative approaches to find HIV-positive people unaware of their HIV status and link them to care and treatment, using both laboratory services and point-of-care (POC) testing.^5^

## HIV-related laboratory infrastructure in Ethiopia for CD4, viral load and early infant diagnosis

**FIGURE 1:**
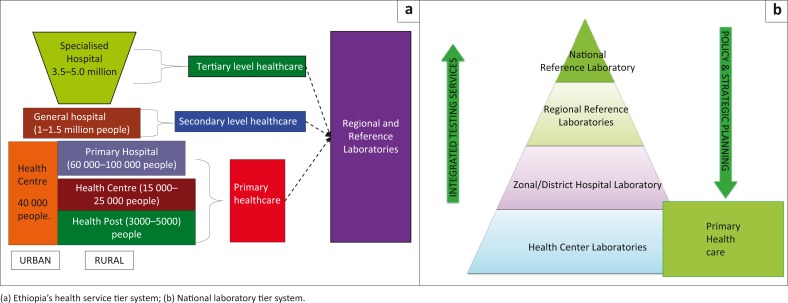
Health system and laboratory network: Ethiopia’s referral linkage guideline.

The public health system in Ethiopia has been decentralised through a tiered laboratory structure. The existing structure at the Federal Ministry of Health enables the federal-level structures to monitor and ensure the effectiveness, efficiency, accessibility, equity and sustainability of health services, as well as engagement of local stakeholders (Figure 1a). The Ethiopian laboratory system comprises a network, including the National Reference Laboratory, Regional Reference Laboratories, and hospital and health centre laboratories, in decreasing order of complexity and testing capacities (Figure 1b). Currently, there are 13 Regional Reference Laboratories with diverse capacities, more than 300 hospital laboratories and 3500 health centre laboratories in the public-health sector. More than 4096 laboratories of various levels are associated with non-governmental organisations or private clinics and hospitals operating throughout the country.^6^

HIV-related testing is one of the most important laboratory services in the health sector. With the launch of a free antiretroviral therapy initiative by the government in January 2005, there has been an unprecedented effort to scale up antiretroviral therapy and provide access to HIV care to all HIV-positive individuals. Currently, there are 10 centres providing DNA PCR for early infant diagnosis of HIV, nine centres for HIV viral load testing using conventional methods and more than 300 centres providing CD4 testing services. The number of centres providing early infant diagnosis and viral load testing using conventional methods grew to 19 by the end of June 2016.

## POC and quality assurance framework in Ethiopia

The Vancouver consensus statement sought the political will and resources needed to meet the new World Health Organization guidelines and related to the UNAIDS 90-90-90 by 2020 goal.^7^ Achieving the UNAIDS goal by increased availability of HIV-associated POC testing devices will improve services. POC testing has been demonstrated to improve linkage to care among persons living with HIV.^8^ Improving antiretroviral therapy coverage quickly and effectively to meet the 90-90-90 goals, by engaging persons living with HIV in care at earlier stages of disease, will require not only expansion of the clinic-based infrastructure with quality-assured POC testing, but also non-clinic-based testing with quality-assured POC testing.^3^

In 2013, the Federal Ministry of Health and the Ethiopian Public Health Institute, with the involvement of stakeholders and partners, developed Ethiopia’s National Strategy for POC test selection, operational evaluation and implementation. As the technical arm of the Ministry, the Ethiopian Public Health Institute is responsible for the selection, operational evaluation and implementation of new diagnostic technologies and is assisted by national technical working groups in the management of the processes. The generic National Strategy document for new POC diagnostic technologies addresses the following important areas: product selection, evaluation, and implementation; supply chain and service/maintenance; quality assurance, connectivity (enables sending of results data from the archive of the Alere Pima™ analyser to a defined central server) and data management; monitoring and evaluation, training and certification of healthcare workers.

The Ethiopian POC testing strategy proposes the adoption of multiple technologies to encourage competition and enhance the deployment of multiple POC testing products in various healthcare settings where they can be effectively utilised. However, it also recommends limiting the number of products evaluated and approved for use in the country, in order to minimise the complexity of managing the supply chain, service and maintenance, training, and monitoring and evaluations.

**FIGURE 2:**
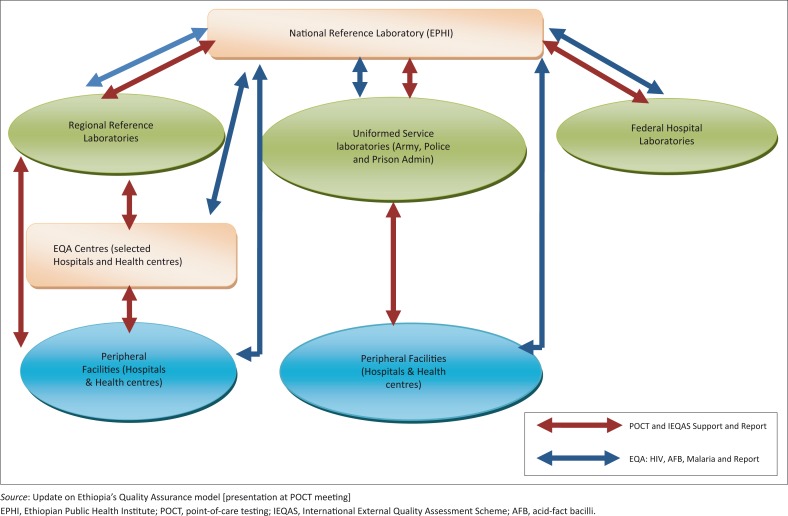
Quality assurance framework.

Quality assurance activities have paramount importance for the effective utilisation of POC testing and must be planned strategically and executed correctly along with the expansion of POC testing, which will require strong support from the Ministry of Health and other stakeholders to all tiers of the national laboratory system (Figure 2).

## Ethiopia’s experiences with implementing POC testing for CD4 and tuberculosis

**FIGURE 3:**
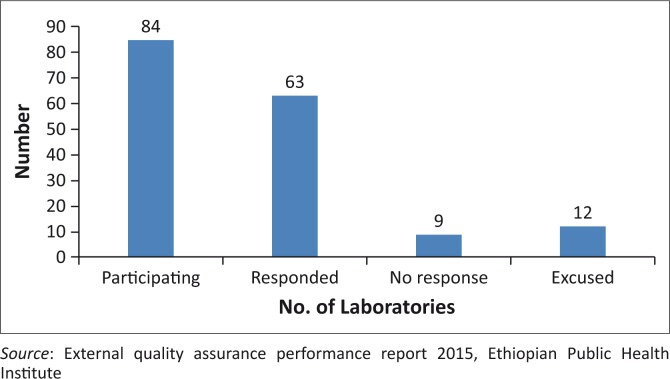
Responses from Pima CD4 sites participating in Oneworld Accuracy test in 2015 (event 2).

After completion of the Alere Pima CD4 evaluation by the Ethiopian Public Health Institute in different regions (unpublished data), 97 units were procured and distributed for scaling up CD4 testing services across the country. The placement of the Pima platforms was guided by strict written criteria, which mainly considered the volume of CD4 testing at facilities. Using this criterion helped effective and efficient placement and use of the devices. Of the 97 Pima platforms, 84 (87.0%) are participating in the Oneworld Accuracy External Quality Assurance (EQA) programme (Health Matrix, Vancouver, Canada). Of these participants, 63 (75.0%) reported proficiency testing results for the second test event of the 2015 cycle (Figure 3). Among laboratories with scores, 75.4% scored 100.0% and root cause analysis and corrective action was undertaken and completed for laboratories with less than 100.0% to complete the quality assurance circle. Nine laboratories did not respond within the deadline, and of the 12 laboratories excused, 8 were excused due to stock outs, one due to electric power outage and three due to specimen transportation issues.

Quality assurance implementation involved training of laboratory personnel at various levels. A five-day training-of-trainers training on Pima technology was provided for regional laboratories, and site-level trainings were organised with certification of trainees. Refresher trainings, including training on laboratory quality management systems, supply chain management and laboratory safety, were also provided. There are currently 104 GeneXpert^®^ (Cepheid) instruments being used for tuberculosis testing in Ethiopia, but only 12 laboratories are participating in the United States Centers for Disease Control and Prevention, International Laboratory Branch EQA proficiency testing programme. Ethiopia has limited experience with connectivity to monitor quality assurance for POC testing. Currently, 70 of the 97 Pima devices are connected. Installation of the GxAlert software application for connectivity on 37 GeneXpert devices is currently underway. For long-term sustainability, plans to build capacity for in-country proficiency testing production through technology transfer to ensure the availability of appropriate EQA methods and programmes for all POC testing sites continues to be an important strategy.

## Existing national quality assurance programmes

### Document standardisation

As part of its quality assurance programme, Ethiopia developed and standardised laboratory diagnosis of malaria, HIV and tuberculosis. In addition, POC implementation, POC testing for CD4 and tuberculosis, national EQA for malaria, guidelines for safety, a manual for sample collection, standard operating procedures for testing, and job aids, among others, were also developed.

### External quality assessment services

**FIGURE 4:**
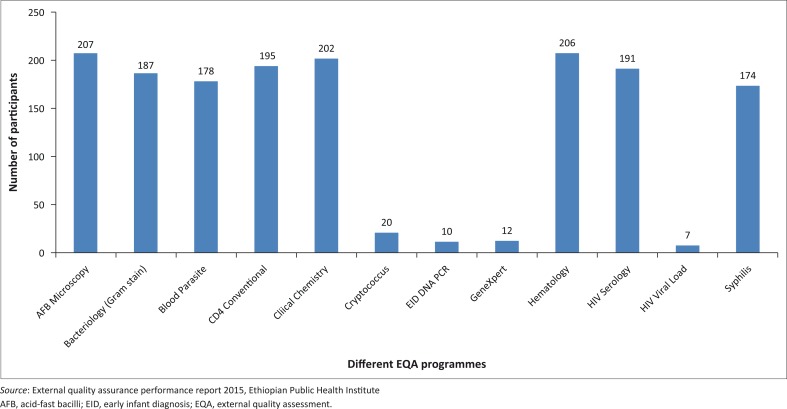
Ethiopian laboratories participating in the national EQA scheme (*Cryptococcus* spp.), international EQA scheme from CDC Atlanta (EID, HIV Viral Load and GeneXpert) and international EQA scheme from Oneworld Accuracy (other tests), March 2016.

In Ethiopia, EQA services are being provided via three schemes (Figure 4). In the international EQA scheme, the Ethiopian Public Health Institute uses testing materials purchased from international EQA service providers. Currently, there are more than 285 laboratories participating in the Oneworld Accuracy EQA system. The national EQA scheme uses testing materials produced and maintained by the National Reference Laboratories for HIV, tuberculosis and malaria. The regional EQA scheme for the same programs is administered by the Regional Reference Laboratories, which are responsible for the production and maintenance of the proficiency testing panels. They also coordinate, conduct and monitor blind rechecking of tests and site supervision within their respective regions. Currently, there are more than 900 facilities with more than 3542 testing points enrolled in the regional EQA scheme for HIV.

### Accreditation

Ethiopia adopted the Stepwise Laboratory Quality Improvement Process Towards Accreditation in 2010 as a tool for laboratory quality improvement. Currently, there are more than 109 laboratories (hospital, regional and national) enrolled in the Stepwise Laboratory Quality Improvement Process Towards Accreditation programme. The Ethiopian Public Health Institute is in the process of assessing the laboratories to select best performers for eventual application for ISO accreditation. Recently, 53 laboratories were assessed with a group of certified auditors from the African Society for Laboratory Medicine; 23 laboratories scored no stars, 11 laboratories scored 1 star, 13 laboratories scored 2 stars, five laboratories scored 3 stars, and one laboratory scored 4 stars.

## Challenges with POC testing

Although Ethiopia has accomplished a great deal in expansion of POC testing, some challenges have been noted. These include: the under-utilisation of Pima and GeneXpert instruments; low EQA coverage for GeneXpert due to shortage of EQA panels; low response rates from proficiency testing participants; supply distribution issues; stock outs; and maintenance issues with prolonged downtime and transportation of devices to maintenance centres.

**Lessons learnt and way forward**

Implementation of POC testing has improved health service delivery through provision of laboratory services with shorter turnaround time. The national quality assurance programme will focus on implementing a comprehensive quality assurance programme for POC testing services that will include strengthening the laboratory–clinic interface, improving the supply chain system through collaboration with supply agencies, ensuring availability of service agreements for all new equipment and building capacity for in-country proficiency testing production through training on and implementation of the ISO/IEC 17043 standard. In collaboration with existing local courier systems, establishing a robust system for panel distribution and result feedback to reach all sites are major areas that will need to also be addressed. In addition, improving implementation of quality POC testing will include strengthening national capacity for ongoing evaluation and validation of new POC testing technologies by supporting the national product selection advisory group. Ethiopia is committed to strengthening both national and regional laboratory capacity through networking with national reference laboratories across the African region for better data sharing that will improve upon systems for monitoring, evaluation and data management, including connectivity.
